# Survival after chemotherapy and/or radiotherapy versus self-expanding metal stent insertion in the setting of inoperable esophageal cancer: a case-control study

**DOI:** 10.1186/1471-2407-12-70

**Published:** 2012-02-15

**Authors:** George Sgourakis, Ines Gockel, Constantine Karaliotas, Markus Moehler, Carl Christoph Schimanski, Heinz Schmidberger, Theodor Junginger

**Affiliations:** 1 Department of General and Abdominal Surgery, Johannes Gutenberg University-Hospital of Mainz, Mainz, Germany; 2 2nd Surgical Department and Surgical Oncology Unit of "Korgialenio-Benakio", Red Cross Hospital, Athens, Greece; 3 1st Medical Clinic and Policlinic, Johannes Gutenberg University-Hospital of Mainz, Mainz, Germany; 4 Department of Radiooncology, Johannes Gutenberg University-Hospital of Mainz, Mainz, Germany; 5 Department of General and Abdominal Surgery, Johannes Gutenberg University-Hospital of Mainz, Langenbeckstr. 1, D-55131 Mainz, Germany

## Abstract

**Background:**

Our aim was to compare survival of the various treatment modality groups of chemotherapy and/or radiotherapy in relation to SEMS (self-expanding metal stents) in a retrospective case-control study. We have made the hypothesis that the administration of combined chemoradiotherapy improves survival in inoperable esophageal cancer patients.

**Methods:**

All patients were confirmed histologically as having surgically non- resectable esophageal carcinoma. Included were patients with squamous cell carcinoma, undifferentiated carcinoma as well as Siewert type I--but not type II - esophagogastric junctional adenocarcinoma. The decision to proceed with palliative treatments was taken within the context of a multidisciplinary team meeting and full expert review based on patient's wish, co-morbid disease, clinical metastases, distant metastases, M1 nodal metastases, T4-tumor airway, aorta, main stem bronchi, cardiac invasion, and peritoneal disease. Patients not fit enough to tolerate a radical course of definitive chemo- and/or radiation therapy were referred for self-expanding metal stent insertion. Our approach to deal with potential confounders was to match subjects according to their clinical characteristics (contraindications for surgery) and tumor stage according to diagnostic work-up in four groups: SEMS group (A), Chemotherapy group (B), Radiotherapy group (C), and Chemoradiotherapy group (D).

**Results:**

Esophagectomy was contraindicated in 155 (35.5%) out of 437 patients presenting with esophageal cancer to the Department of General and Abdominal Surgery of the University Hospital of Mainz, Germany, between November 1997 and November 2007. There were 133 males and 22 females with a median age of 64.3 (43-88) years. Out of 155 patients, 123 were assigned to four groups: SEMS group (A) n = 26, Chemotherapy group (B) n = 12, Radiotherapy group (C) n = 23 and Chemoradiotherapy group (D) n = 62. Mean patient survival for the 4 groups was as follows: Group A: 6.92 ± 8.4 months; Group B: 7.75 ± 6.6 months; Group C: 8.56 ± 9.5 months, and Group D: 13.53 ± 14.7 months. Significant differences in overall survival were associated with tumor histology (*P *= 0.027), tumor localization (*P *= 0.019), and type of therapy (*P *= 0.005), respectively, in univariate analysis. Treatment modality (*P *= 0.043) was the only independent predictor of survival in multivariate analysis. The difference in overall survival between Group A and Group D was highly significant (*P *< 0.01) and in favor of Group D. As concerns Group D versus Group B and Group D versus Group C there was a trend towards a difference in overall survival in favor of Group D (*P *= 0.069 and *P *= 0.059, respectively).

**Conclusions:**

The prognosis of inoperable esophageal cancer seems to be highly dependent on the suitability of the induction of patient-specific therapeutic measures and is significantly better, when chemoradiotherapy is applied.

## Background

Accurate information regarding the proportion of patients with esophageal cancer in whom surgery is contraindicated is difficult to obtain. This largely reflects variations in the selection of patients for palliative treatment modalities. In the Western world, more than half the patients with esophageal cancer are not amenable to surgery as they usually present with severe comorbidity and an advanced stage of disease [1].

The choice of treatment must be tailored to the individual and will depend on the location and stage of the tumor, as well as the overall health of the patient.

Four RCT's [2-5] and one meta-analysis [6] compared brachytherapy, laser ablation therapy and argon beam coagulation (APC) therapy with self-expanding metal stents within the context of esophageal cancer palliation. The aforementioned studies present symptomatic patient relief as the primary outcome and patient survival as the secondary. Only one of the studies [3] provides data for external beam radiation therapy, but patients are collectively analyzed with those who underwent APC.

It has also been suggested that combination chemoradiotherapy may improve response rates and thus survival, although evidence is limited [7]. A study providing a straightforward comparison between chemotherapy and/or radiotherapy and SEMS is lacking.

We have made the hypothesis that the administration of combined chemoradiotherapy improves survival in inoperable esophageal cancer patients. Our aim was to specify survival of the various treatment modalities in relation to SEMS in a retrospective case-control study.

## Methods

From November 1997 to November 2007, a total of 437 patients presented to our institution with histologically proven esophageal carcinoma. Esophagectomy was contraindicated in 155 (35.5%) patients (133 males, 22 females) with a median age of 64.3 (43-88) years. This represents a group of individuals for whom a minimum of 4 years of follow-up data was possible.

Reasons of incurability were distant metastases (n = 54; 34.8%), local tumor spread (n = 58; 37.4%) and preexistent cardiopulmonary diseases (n = 26; 16.8%). Seventeen (11%) patients presented further reasons of incurability. Of these, 5 patients refused surgery, and 5 were excluded from surgery as they did not have an adequate substitute organ for reconstruction and esophagoplasty. In 7 patients, the risk of esophagectomy was considered too high due to their poor general health status.

After histological confirmation, all patients underwent a preoperative diagnostic work-up, in addition to computed tomography of the neck, thorax, and abdomen, which included endosonography of the esophagus, barium swallow, transabdominal sonography of the abdomen, as well as positron emission tomography (PET), as previously described [8]. A conventional X-ray examination of the thorax and laboratory tests with the tumor markers carcinoembryonic antigen (CEA), Ca 19-9, Ca 72-4, and Alpha-Feto-Protein (AFP) were routinely performed.

### Inclusion and exclusion criteria

All patients were confirmed histologically as having esophageal carcinoma. Included were patients with squamous cell carcinoma (SCC), undifferentiated carcinoma (UDC), as well as Siewert type I--but not type II--esophagogastric junctional adenocarcinoma (ADC) [9].

The decision to proceed with palliative treatments was taken within the context of a multidisciplinary team meeting and full expert review based on patient's wish, co-morbid disease, distant metastases, M1 nodal metastases, and T4 tumor.

Hematogenic and lymphogenic metastases were documented by computed tomography (CT) examination, PET scan and endosonographic ultrasound. In summary, a CT diagnosis of T4-tumor stage made an R0 resection impossible.

Standard indicators defining a patient as medically inoperable included baseline forced expiratory volume in the first second of expiration (FEV1) of less than 40% predicted, carbon monoxide diffusing capacity of less than 40% predicted, baseline hypoxemia or hypercapnia, severe pulmonary hypertension; diabetes mellitus with end-organ damage; severe cerebral, cardiovascular, or peripheral vascular disease; or severe, chronic heart disease.

### Multidisciplinary team strategy

Patients not fit enough to tolerate a radical course of definitive chemotherapy and/or radiation, or those who needed rapid relief of their dysphagia (swallowing may deteriorate because of radiation induced edema and swelling of the tumor) were referred for self-expanding metal stent (SEMS) insertion. Radiochemotherapy was carried out according to the Herskovic protocol (four courses of combined fluorouracil and cisplatin plus 5,000 cGy of radiation therapy) [10]. In brief, patients without distant metastases underwent external beam radiotherapy with a dose of 50-60 Gy and, whenever possible, an additional brachytherapy of up to 68 Gy. In selected patients with previous cancer and consecutive radiotherapy (e.g. oropharyngeal, laryngeal cancer, etc.), these doses had to be individually adapted. Patients receiving chemotherapy alone were given a combination of fluorouracil and cisplatin, providing no contraindications were present. Patient groups were not randomized.

Locally inoperable squamous cell carcinoma was treated by chemoradiotherapy, radiation therapy alone, or palliative chemotherapy in cases of distant metastases, whereas advanced or metastasized adenocarcinoma was treated by chemotherapy or chemoradiotherapy.

### Control for confounding variables

Data of patients with esophageal carcinoma undergoing the four main treatment modalities (SEMS, chemotherapy and/or radiotherapy) were matched according to clinical characteristics (contraindications for surgery) and tumor stage by diagnostic work-up.

The confounding variables were chosen according to the Standardization or Adjustment method: we applied the cumulative proportion surviving rates generated in each of our four strata (stent, chemotherapy, radiotherapy, chemoradiotherapy) to the same "standard" theoretical population. This standard population was created so that the frequency of the confounder is identical between each group of patients; this was achieved by applying sequential filters to the Microsoft Excel database.

According to their treatment modality, patients were assigned to four groups: SEMS group (A), Chemotherapy group (B), Radiotherapy group (C), and Chemoradiotherapy group (D).

According to the study hypothesis, the control group included patients in whom a self expandable stent had been inserted.

### Definitions

In patients with histologically proven esophageal carcinoma, the following prognostic variables were recorded, American Society of Anesthesiologists (ASA) classification (I-IV) according to the anesthesiology evaluation, body mass index (BMI) based on body weight and height in kg/m^2^, and the nutritional status including tobacco and/or alcohol abuse. Tobacco abuse was defined by the consumption of at least 5 cigarettes a day over a period of > 1 year, whereas alcohol abuse was defined by the regular intake of beer, wine or hard drinks, at least every second day. Among the comorbidities, cardiovascular risk factors were defined as a history of coronary heart disease, or myocardial infarction, arterial hypertension, valvular disease (> II°), arrhythmia requiring therapy (> III° according to the Lown classification), heart failure NYHA (New York Heart Association) > grade II, and peripheral occlusive arterial disease (> IIb according to Fontaine). A history of chronic obstructive pulmonary disease (COPD), regular tobacco consumption, and/or the use of bronchospasmolytics were subsumed under pulmonary diseases. The preoperative evaluation of the vital capacity (VC) and the forced expiratory volume in 1 s (FEV1 = Tiffeneau test) served to ensure a more accurate assessment. Preexisting cirrhosis of the liver (≥CHILD-Pugh A) was defined as hepatic disease, and determined on the basis of the measurement of serum albumin (g/dl), serum bilirubin (mg/dl), Quick value (%), and the presence of ascites or encephalopathy. The evaluation of additional risk factors included the prevalence of diabetes mellitus (insulin-dependent or requiring drug therapy), and the history of a secondary carcinoma.

For a better comparison of staging procedures, the esophagus was considered in thirds, according to the endoscopic location of the tumor: upper third: dental front to 20 cm; middle third: 20-30 cm; lower third: 30 cm to the Z-line.

The various study parameters were coded as follows: ***Procedures: ***1 = explorative laparotomy/laparoscopy, but no resection; 2 = none; 3 = primarily neoadjuvant intention; 4 = surgical exploration, but no resection; 5 = explorative thoracotomy/thoracoscopy, but no resection. ***Tumor histology: ***1 = squamous cell cancer; 2 = adenocarcinoma; 3 = undifferentiated carcinoma. ***Contraindication for surgery: ***1 = metastases; 2 = local tumor spread; 3 = cardiopulmonary; 4 = pulmonary; 5 = cardiac; 6 = esophageal substitute not available; 7 = tumor spread and poor general condition; 8 = tumor spread and cardial disease; 9 = patient refuses surgery; 10 = mucosal resection; 11 = metastases and poor general condition; 12 = patient refuses surgery and poor general condition. ***Reasons for non-surgical management: ***1 = metastases; 2 = locally not resectable; 3 = cardiopulmonary contraindication; 4 = other. ***Metastasis site: ***1 = liver; 2 = lung; 3 = lung + liver; 4 = M1 lymph nodes; 5 = diffuse; 6 = peritoneal carcinosis; 7 = bone; 8 = brain; 9 = skin. ***Type of metastases: ***1 = hematogeneous; 2 = lymphatic. ***Treatment modality: ***1 = stent; 2 = chemotherapy; 3 = radiation; 4 = radio-chemotherapy; 5 = tracheostomy; 6 = mucosectomy. For all clinical characteristics, the presence of the variables mentioned was declared 1, and the absence 0.

Approval for the study was obtained from the hospital ethics committee.

### Statistical analysis

In order to validate our patient selection with confounding variables, two different statistical tests were applied: 1) Kruskal-Wallis ANOVA by Ranks test (Median test) was used in order to compare the clinico-pathological parameters (numerical) of patients among the various groups of therapy and compute post-hoc comparisons of mean ranks of all pairs attributed to by groups and 2) Spearman's correlations were also used to analyze the distribution of the clinico-pathological parameters (coded in categories) separately in each group of therapy.

A multivariate analysis was performed under Cox's Proportional Hazard Model considering all factors that gained statistical significance (*P *< 0.05) in univariate analysis under the same model. Variables that reached significance in the multivariate model were considered as predictors of survival. In order to compare survival between two samples, the Cox's F-test was employed. So as to validate our results in terms of cumulative proportion surviving, sample size calculation was performed with a type I error rate (Alpha) 0.05 and power goal 0.80 by applying the two-tailed Log-Rank test.

Significance was considered at a level < 0.05. Statistical release 7 (Statsoft, Tulsa, USA) was used for statistical analysis.

## Results

In relation to all patients presenting with esophageal cancer from November 1997 to November 2007, the proportion of inoperable patients was higher in SCC's (23.8%; n = 104/437) as compared to ADC's (10.5%; n = 46/437) (in addition 5 patients with UDC). Both tumor entities revealed different reasons of inoperability: In patients with SCC, local tumor spread predominated with 45.7%, whereas in the majority of patients with ADC (58.7%), inoperability was due to hematogeneous metastases. Cardiopulmonary diseases causing contraindication for surgery were equally distributed among both tumor entities (17.1% in patients with SCC and 17.4% in patients with ADC).

The course of the disease was ascertained in 152 out of 155 (98%) patients by December 31st 2010; no data documenting the course were available in 3 patients at that time. One hundred and forty-eight patients had died from their malignancy; of the remaining 7 patients, 4 were alive and 3 were lost from follow-up. All 4 patients alive at the time of last follow-up had initially presented with a locally inoperable SCC and had undergone radiochemotherapy.

Six patients who underwent mucosectomy (n = 2) and tracheostomy (n = 1) or were lost from follow-up (n = 3) were not included in the analysis. The respective numbers of patients for SCC, ADC and UDC were 104, 46 and 5. According to the diagnostic work-up, tumor stage was IIA in 17 patients, IIB/III in 76 patients and IV in 54 patients. Contraindications for surgery were cardiopulmonary status in 26 patients, metastasis and tumor spread in 119, surgery refusal by 5, and no chance of esophageal substitution in five patients.

Brachytherapy was administered to 2 patients in group C and to 16 patients in group D. Seven patients in group B, 10 in group C, and 37 patients in group D completed their allocated treatment modality.

UDC patients were excluded since there were missing data in 3 (tumor stage), while the remaining 2 patients were excluded during the selection process.

**Figure 1:**
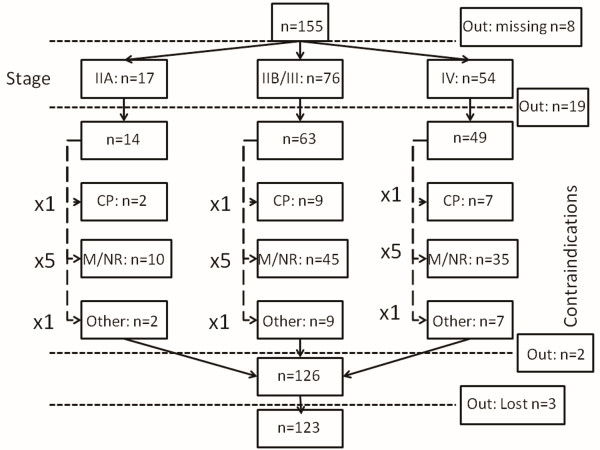
**Only patients without missing data (in terms of the confounding variables) were included by applying sequential filters to the Microsoft Excel database**. Contraindications for surgery were divided into 3 categories: **a**) cardiopulmonary (CP), **b**) metastasis/non-resectable (M/NR) and c) other. Patients were considered accordingly by an analogy 1/5/1 (paralleling the rates among the 155 patients) in each of the 3 patient classes of the TNM-stage (IIA, IIB/III, IV) which were considered respectively by an analogy of 2/9/7 (paralleling the rates among the 155 patients). These sequential steps reduced the total 155 patients by 8 patients (missing data), 19 patients by "TNM-stage" filtering, 2 patients by "contraindications for surgery" filtering and by 3 patients lost to follow up. The remaining 123 patients were assigned to four groups.

In applying the sequential filters for confounding variables, only 123 out of 155 patients were considered for the analysis. We took care to include only patients without missing data, in terms of contraindications for surgery and TNM-stage by applying sequential filters to the Microsoft Excel database (Figure 1). Contraindications for surgery were divided into 3 categories: a) cardiopulmonary, b) metastasis/non-resectable and c) other. Patients were considered accordingly by an analogy 1/5/1 (paralleling the rates among the 155 patients) in each of the 3 patient classes of the TNM-stage (IIA, IIB/III, IV) which were considered respectively by an analogy of 2/9/7 (paralleling the rates among the 155 patients) (Figure 1). These sequential steps reduced the total of 155 patients by 8 patients (missing data), 19 patients by "TNM-stage" filtering, 2 patients by "contraindications for surgery" filtering and 3 patients lost to follow up (Figure 1). The remaining 123 patients were assigned to four groups: SEMS group (A) n = 26 (SCC:10/ADC:16), Chemotherapy group (B) n = 12 (SCC:10/ADC:2), Radiotherapy group (C) n = 23 (SCC:11/ADC:12), Chemoradiotherapy group (D) n = 62 (SCC:52/ADC:10). For every patient in group B, approximately 2 patients in groups A and C, and 5 in group D were analyzed in terms of survival.

As concerns the allocation of clinico-pathological characteristics among the 4 groups, all 21 parameters were equally distributed in terms of age, tumor localization, type of metastasis, histology, and cardiopulmonary status (Table 1).

**Table 1:** Comparison of clinico-pathological characteristics among the 4 groups of treatment modality

Variable (continuous)		Kruskal-Wallis ANOVA by Ranks test (Median test) Mean ± SD	*P*-value
**Age**	**A SEMS** **B Chemo** **C Radiation** **D C/R**	69.57 ± 10.08	***P = 0.017 ****(*SEMS vs. C/R* *P *= 0.013)
		67.00 ± 4.30	
		66.44 ± 8.57	
		61.05 ± 9.74	
		
**BMI**		24.87 ± 4.27	*P *= 0.466
		25.91 ± 5.65	
		25.70 ± 4.00	
		24.81 ± 5.11	
		
**Vital capacity (VC)**		3.54 ± 1.08	*P *= 0.287
		3.75 ± 0.93	
		2.75 ± 1.28	
		3.78 ± 0.95	
		
**FEV**_**1**_		32.53 ± 33.53	*P *= 0.306
		41.29 ± 33.42	
		38.86 ± 30.13	
		46.98 ± 28.11	

**Variable (categorical)**	**Distribution of variable categories among groups of therapy**	**Spearman Rank Order Correlations**	***P*-value**
		
**cTNM-classification**		-0.124360	0.165
		
**ASA-classification**		-0.017661	0.876
		
**Nutrition**		0.166002	0.244
		
**Smoking**		0.080980	0.387
		
**Alcohol**		0.082105	0.380
		
**Child-Pugh score**		0.033203	0.724
		
**Pulmonary status**		0.226517	***0.013***
		
**Cardiovascular status**		-0.226854	***0.013***
		
**Diabetes**		-0.043337	0.642
		
**Secondary carcinoma**		0.095611	0.286
		
**Gender**		-0.146058	0.095
		
**Histology**		-0.348704	***0.001***
		
**Localization**		-0.259496	***0.002***
		
**Contraindications for surgery**		0.032170	0.715
		
**Causes of inoperability**		0.128822	0.142
		
**Site of metastases**		-0.060909	0.566
		
**Type of metastases**		0.224707	***0.032***

*SD*: Standard deviation; *SEMS*: Self-expandable metallic stents; C/R*: Chemo-Radiotherapy; *BMI*: body mass index; *ASA*: American Society of Anesthesiologists classification; *FEV_1_*: Forced expiratory volume in 1 s

Overall median and mean patient survival was 6 months and 10.89 ± 12.63 months, respectively. The respective numbers for the 4 groups were as follows: Group A: 3/6.92 ± 8.4 months; Group B: 7/7.75 ± 6.6 months; Group C: 4/8.56 ± 9.5 months and Group D: 8/13.53 ± 14.7 months. Overall survival for the 4 groups is depicted in Table 2.

**Table 2:** Cumulative proportion surviving of the 4 groups

Months	Chemo-radiotherapy	Chemotherapy	Radiation	Stent
6	60.60%	43.47%	58.33%	38.46%

12	39.39%	34.78%	25.00%	19.23%

18	30.30%	13.04%	08.33%	07.69%

24	24.24%	08.69%	0%	03.84%

30	10.60%	04.34%		01.92%

36	07.57%	02.17%		00.96%

42	06.06%	0%		0%

48	05.19%			

54	04.32%			

60	03.46%			

Twenty-one variables were analyzed in univariate analysis of survival: gender, age, BMI, vital capacity (VC), FEV_1_, cTNM-classification, ASA-classification, nutritional status, tobacco consumption, alcohol intake, diabetes, Child-Pugh score, cardiopulmonary status, existence of a second carcinoma, histopathology, tumor localization, contraindication for surgery, cause of inoperability, site of metastasis and type of metastasis. Significant differences in overall survival were associated with tumor histology (*P *= 0.027), tumor localization (*P *= 0.019), and type of therapy (*P *= 0.005), respectively (Table 3).

**Table 3:** Univariate analysis of survival

	Beta	Wald statistic	*P*-value*
**ASA-classification**	-0.034372	0.034097	0.853501

**Nutrition status**	-0.059647	0.310746	0.577226

**Smoking**	-0.282645	2.403644	0.121063

**Alcohol consumption**	-0.294566	2.635858	0.104485

**Hepatopathy**	0.066506	0.091186	0.762676

**Pulmonary disease**	-0.062020	0.114867	0.734672

**Cardial disease**	-0.276026	2.215080	0.136678

**Diabetes**	-0.241763	1.005458	0.316001

**Secondary carcinoma**	0.196181	0.924205	0.336381

**Vital Capacity (VC)**	-0.239513	2.552092	0.110158

**FEV**_**1**_	-0.296171	3.468683	0.062550

**Age**	0.008591	0.774818	0.378737

**Gender**	0.319681	1.808111	0.178745

**Procedure**	0.081361	0.364907	0.545798

**Histology**	0.362451	4.884958	**0.027099**

**Localization**	0.241270	5.437840	**0.019711**

**Contraindication** **for surgery**	-0.041959	1.555456	0.212341

**Causes of incurability**	-0.053454	0.340351	0.559631

**Site of metastases**	0.087241	1.488161	0.222511

**Type of metastases**	-0.346926	2.826217	0.092746

**Therapy**	-0.199524	7.805245	**0.005213**

**BMI**	-0.003725	0.050582	0.822056

*P-values in bold depict statistical significance

*BMI*: body mass index; *ASA*: American Society of Anesthesiologists classification; *FEV_1_*: Forced expiratory volume in 1 s

In multivariate Cox's Proportional Hazard regression analysis of survival, the model including the predictors in univariate analysis gained statistical significance (*P *< 0.001), but treatment modality (*P *= 0.043) was the only independent predictor of survival (Table 4).

**Table 4:** Multivariate analysis of survival

	Beta	Wald statistic	*P*-value*
**Histology**	0.077891	0.142777	0.705539

**Localisation**	0.167822	1.988769	0.158479

**Type of therapy**	-0.157421	4.059833	**0.043923**

*P-values in bold depict statistical significance

With regard to the 12-month hazard rate/standard error of hazard rate for the 4 treatment groups: Group A = 0.14/0.07, Group B = 0.16/0.10, Group C = 0.15/0.06, and Group D = 0.04/0.01.

**Figure 2:**
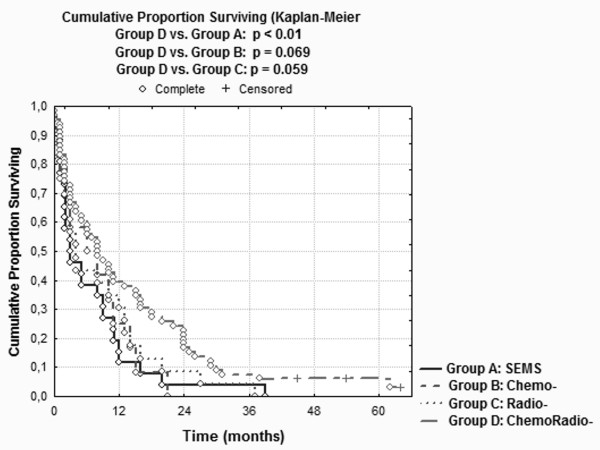
**Cumulative proportion surviving for the patients of the 4 groups**.

The difference in overall survival between Group A and Group D was highly significant (*P *< 0.01), and in favor of Group D. Comparing Group D versus Group B and Group D versus Group C, the difference in overall survival was marginally significant, and in favor of Group D (*P *= 0.069 and *P *= 0.059, respectively). The other possible comparisons in overall survival between Groups did not reach statistical significance (Figure 2).

Aiming to establish a 2-year difference in overall survival of 24.3% vs. 3.8% for groups D and A respectively, there was a need for 26 patients per treatment group. The respective number for group D vs. C was 23 patients (24.3% vs. 0%), and for group D vs. B, it was 61 patients (24.3% vs. 8.6%). All the aforementioned comparisons of overall survival between groups fulfilled the sample size calculation, with the exception of one (group D: 62 patients vs. group B: 12 patients).

In order to investigate the rationale of our multidisciplinary team strategy to assign more adenocarcinoma patients to chemotherapy alone (n = 12) than to chemoradiotherapy (n = 9), survival curves of these two groups were compared. No statistical significant difference in overall survival was observed (*P *= 0.589). The same applied to squamous cell carcinoma patients undergoing chemoradiotherapy (n = 55) versus radiotherapy alone (n = 10) (*P *= 0.405).

## Discussion

In the setting of care in inoperable esophageal cancer, the combination of chemoradiotherapy was more efficacious than any other treatment modality, and undoubtedly superior to the use of SEMS in terms of patient survival. Only chemoradiation patient Group D had 4- and 5-year survivors.

Though the clinico-pathological characteristics among patients of the 4 groups were not significantly different, there were distinct differences observed in some. This is mainly attributed to the multidisciplinary team strategy: 1) Patients not fit enough to tolerate a radical course of definitive chemotherapy and/or radiation were referred for self-expanding metal stent insertion, and 2) Patients with locally inoperable squamous cell carcinoma were treated by chemoradiotherapy, radiation therapy alone or chemotherapy in cases of distant metastases, whereas patients with advanced adenocarcinoma were treated by chemotherapy or chemoradiotherapy. Despite this potential patient selection bias, survival curves of the aforementioned groups were not significantly different.

Although various studies [9,11] have described histologic differentiation as an independent prognostic factor after R0 resection, our results showed no evidence of significance supporting this fact in inoperable esophageal cancer. The only decisive predictor of survival was the type of treatment modality as shown in our multivariate analysis. However, in cases of rather favorable prognosis among inoperable patients, palliation *and *survival have to be considered, whereas in individuals with rather unfavorable findings and a projected limited survival, palliation should be the main focus. This includes rapid and effective alleviation of symptoms as well as avoidance of possible complications and repeated interventions, thus improving or maintaining patients' quality of life.

Our study, involving an inoperable patient cohort, yielded similar results to those of other prospective studies [12,13] and a meta-analysis of randomized trials [14], which evaluated combined chemoradiotherapy versus radiotherapy alone in patients with localized esophageal carcinoma.

Unlike our data with four long-term survivors, who had initially presented with locally inoperable cancer, better survival following radiochemotherapy has been reported in the literature for patients primarily excluded from surgery due to preexistent comorbidity [15]. In some studies, however, life-expectancy was unfavorable in patients with cT4-tumors [14,15].

This study was conducted, despite the risk of selection bias, since a randomized controlled trial resolving the issue is hardly, if ever, feasible. It is not without its drawbacks, mainly: 1) It is not a prospective randomized, but a retrospective case-control study; 2) The fact, that cT-, cN- and cM-categories in inoperable disease were proven by CT, endosonographic ultrasound, PET scan, and in selected patients by staging laparoscopy/laparotomy or thoracoscopy/thoracotomy, may be a source of bias because clinical and histological stage may differ; 3) Other established treatment modalities, such as brachytherapy only (without combined radiation therapy) or Argon beam coagulation were not included as main or accessory measures for palliative treatment, although these did not form part of the research question; 4) Even though patient data was matched to deal with the confounders of contraindications to surgery and stage of disease, the fact that patients received a stent because they were not able to tolerate chemoradiotherapy and had significant dysphagia can potentially affect the outcome; 5) Patients' symptom relief and procedural complications were not included due to incomplete data, but this issue has already been resolved by a recent meta-analysis [6].

## Conclusions

In summary, the prognosis of inoperable esophageal cancer seems to be highly dependent on the suitability of the induction of patient-specific therapeutic measures and is better when chemoradiotherapy is applied, though this is not proven by randomized data in our patient cohort.

## Competing interests

The authors declare that they have no competing interests.

## Authors' contributions

GS, IG, and TJ conceived of the study, analysed the results and drafted the manuscript. MM and CCS carried out the chemotherapy, and HS the irradiation of patients. GS, IG, CK, and TJ participated in the design of the study and performed the statistical analysis. All authors read and approved the final version of the manuscript.

## Pre-publication history

The pre-publication history for this paper can be accessed here:

http://www.biomedcentral.com/1471-2407/12/70/prepub
